# Supplementary material for: Periodontal Bacteria and Outcomes Following Aneurysmal Subarachnoid Hemorrhage: A Prospective Observational Analysis
Source: Biomedicines. 2025 Dec 25;14(1):48. doi: 10.3390/biomedicines14010048 (PMC12837805; doi:10.3390/biomedicines14010048)
Supplement: Supplementary file 1 [file biomedicines-14-00048-s001.zip › biomedicines-3996943-supplementary.pdf]

Supplement Table S1. Isolated bacterial species in healthy controls

Isolated species	n	PPD
Veillonella parvula	8	PPD <5 mm
Veillonella atypica	4	PPD <5 mm
Gemella sanguinis	1	PPD <5 mm
Bacteroides fragilis	1	PPD <5 mm
Pseudoleptotrichia goodfellowi	2	PPD <5 mm
Fusobacterium nucleatum	6	PPD <5 mm, except 2 cases
Porphyromonas somerae	1	PPD <5 mm
Porphyromonas gingivalis	2	PPD <5 mm, except 1 case
Prevotella buccae	3	PPD <5 mm
Actinom.naesl	2	PPD <5 mm
Peptostr.stom.	1	PPD <5 mm
Prevotella melaninogenica	1	PPD <5 mm
Fusobacterium varium	1	PPD <5 mm
Capnocythophaga sputigena	1	PPD <5 mm
Granulicatella adiacens	2	PPD <5 mm
Schaalia odontolyticus	3	PPD <5 mm
Bifidobacterium dentium	3	PPD <5 mm
Lactobacillus sp	1	PPD <5 mm
Pepetoniphilus stomatis	1	PPD <5 mm
Parvimonas micra	2	PPD <5 mm
Actinomyces oris	1	PPD <5 mm
Fusob.gan	1	PPD <5 mm
Eikenella corrodens	1	PPD <5 mm
Veillonella rogosae	1	PPD <5 mm

n, number of healthy subjects positive for the corresponding bacterial species;
PPD, periodontal pocket depth.
